# The effect of national lockdown due to COVID-19 on emergency department visits

**DOI:** 10.1186/s13049-020-00810-0

**Published:** 2020-12-04

**Authors:** Ilari Kuitunen, Ville T. Ponkilainen, Antti P. Launonen, Aleksi Reito, Teemu P. Hevonkorpi, Juha Paloneva, Ville M. Mattila

**Affiliations:** 1 University of Eastern Finland, School of Medicine, Yliopistonranta 1, PL 1627, 70211 Kuopio, Finland; 2 Mikkeli Central Hospital, Porrassalmenkatu 35-37, 50100 Mikkeli, Finland; 3 Department of Orthopaedics and Traumatology, Tampere University Hospital, Teiskontie 35, PL2000, 33521 Tampere, Finland; 4 Department of Surgery, Central Finland Hospital, Keskussairaalantie 19, 40620 Jyväskylä, Finland; 5 Faculty of Medicine and Health Technology, Tampere University, Tampere, Finland

**Keywords:** COVID-19, Pandemic, Epidemiology, Emergency medicine, Stroke, Acute myocardial ischaemia

## Abstract

**Background:**

COVID-19 outbreak lead to nationwide lockdown in Finland on the March 16th, 2020. Previous data regarding to the patient load in the emergency departments during pandemics is scarce. Our aim is to describe the effect of national lockdown and social distancing on the number and reasons for emergency department (ED) visits and inpatient admissions in three large volume hospitals prior to and after the outbreak of the COVID-19 epidemic in Finland.

**Methods:**

Data for this register-based retrospective cohort study were collected from three large ED’s in Finland, covering 1/6 of the Finnish population. All patients visiting ED’s six weeks before and six weeks after the lockdown were included. Pediatric and gynecological patients were excluded. Numbers and reasons for ED visits and inpatient admissions were collected. Corresponding time period in 2019 was used as reference.

**Results:**

A total of 40,653 ED visits and 12,226 inpatient admissions were analyzed. The total number of ED visits decreased 16% after the lockdown, whereas the number of inpatient admissions decreased 15% (*p* < 0.001). This change in inpatient admissions was similar in all participating hospitals. Visits due to back or limb pain decreased 31% and infectious diseases 28%. The visit rate and inpatient admissions due to acute myocardial infarction and strokes remained stable throughout the study period. Interestingly, the rate of inpatient admissions due to psychiatric diagnoses remained unchanged, although the ED visit rate decreased by 19%. The number of ED visits (*n* = 282) and inpatient admissions (*n* = 55) due to COVID-19 remained low in the participating hospitals.

**Conclusions:**

Changes in ED visits and inpatient admissions prior to and during the early phase of the COVID-19 outbreak were unpredictable, and our results may help hospitals and especially ED’s focus their resources better. Surprisingly, there was a major decrease in the rate of ED visits due to back or limb pain and not so surprisingly in infectious diseases. Rates of acute myocardial infarctions and cerebral strokes remained stable. In summary, stabile resources for the treatment of patients with severe diseases will be needed in hospitals and ED’s.

## Background

The coronavirus disease (COVID-19) outbreak was first described in Wuhan, China in December 2019 [1]. Thereafter, the disease spread rapidly, first in China and later globally [2–4]. On March 12, 2020, the World Health Organization (WHO) declared COVID-19 a pandemic [5]. In Finland, the first COVID-19 case was diagnosed on January 28 [6]. By March 16, the cumulative number of cases in Finland was 272, and the Finnish Government declared a state of emergency for the first time since World War II [7, 8].. Measures, such as prohibiting gatherings of more than 10 persons, closing borders, and quarantining residents re-entering Finland for 14 days, were introduced. Public institutions (schools, libraries) were closed, and inhabitants 70 years and older were asked to self-isolate. Moreover, hospitals cancelled elective surgical operations and prepared for the expected arrival of patients with COVID-19. In addition, medical personnel were re-educated for respiratory nursing and hospital resources were focused on COVID-cohorts and ICUs [8].

The rationale behind the national lockdown was that social distancing could impede the spread of the disease. Indeed, it has been suggested that social distancing might have reduced the spread of severe acute respiratory syndrome (SARS) in 2003 and H1N1 influenza in 2009 [9, 10]. Moreover, social distancing seemed to stop the spread of SARS-CoV-2 in China [11–13]. It can, however, be hypothesized that social distancing and the fear of contracting COVID-19 may also increase the threshold to seek medical assistance in other diseases. To date, the published literature regarding how social distancing and a state of national emergency affects the general in-hospital patient load and the patient material of the emergency department (ED) is scarce.

The aim of our study is therefore to describe the effect of national state of emergency measures and social distancing on the number of emergency department (ED) visits, diagnoses, and inpatient admissions in three large volume hospitals prior to and during the early phase of the COVID-19 epidemic in Finland.

## Methods

Three large Finnish hospitals - Tampere University Hospital (TAUH), Central Finland Hospital (CFH), and Mikkeli Central Hospital (MCH) – participated in this retrospective study. In total, the combined catchment population of the three hospitals is 900,000, or 1/6 of the Finnish population [14]. TAUH is a tertiary level hospital with approximately 100,000 ED visits annually. CFH is the largest secondary level hospital in Finland, with 85,000 ED visits per year, and MCH has 50,000 ED visits per year. The study period started six weeks prior to and ended six weeks after the implementation of the lockdown (February 1, 2020 to April 30, 2020). Information on visits, diagnoses, and inpatient admissions for ED patients were collected from the electronic medical databases of the three hospitals. We included all patients aged 15 or over, but we excluded gynecological patients, who are treated in separate EDs in Finland. As a reference, we used data from the corresponding dates in 2019.

General mobility during the study period was evaluated using open-access data from the Finnish Transport Infrastructure Agency [15]. Moreover, the number of vehicles passing the automatic measurement points on the main roads of the participating cities was analyzed daily. The numbers were used as an external reference to present the general effect of the lockdown restrictions.

The present study received research permission from each of the participating hospitals. According to Finnish law, an ethical committee statement is not required for studies using anonymous register data.

### Statistical analysis

The total rates of ED visits, diagnoses, and inpatient admissions before and after the declaration of the state of emergency were evaluated and compared with the rates from the same time period in 2019 to describe the difference from the rate in the previous year. Diagnoses of the visits were classified based on ICD-10 classifications and were analyzed in subgroups based on common reasons for ED visits. Medians and interquartile ranges were analyzed for continuous nonparametric variables and tested by Mann Whitney U – test. Categorized variables were analyzed by crosstabulation and tested by chi square test. All calculations were performed using R version 3.6.2 (R Foundation for Statistical Computing, Vienna, Austria).

## Results

**Fig. 1:**
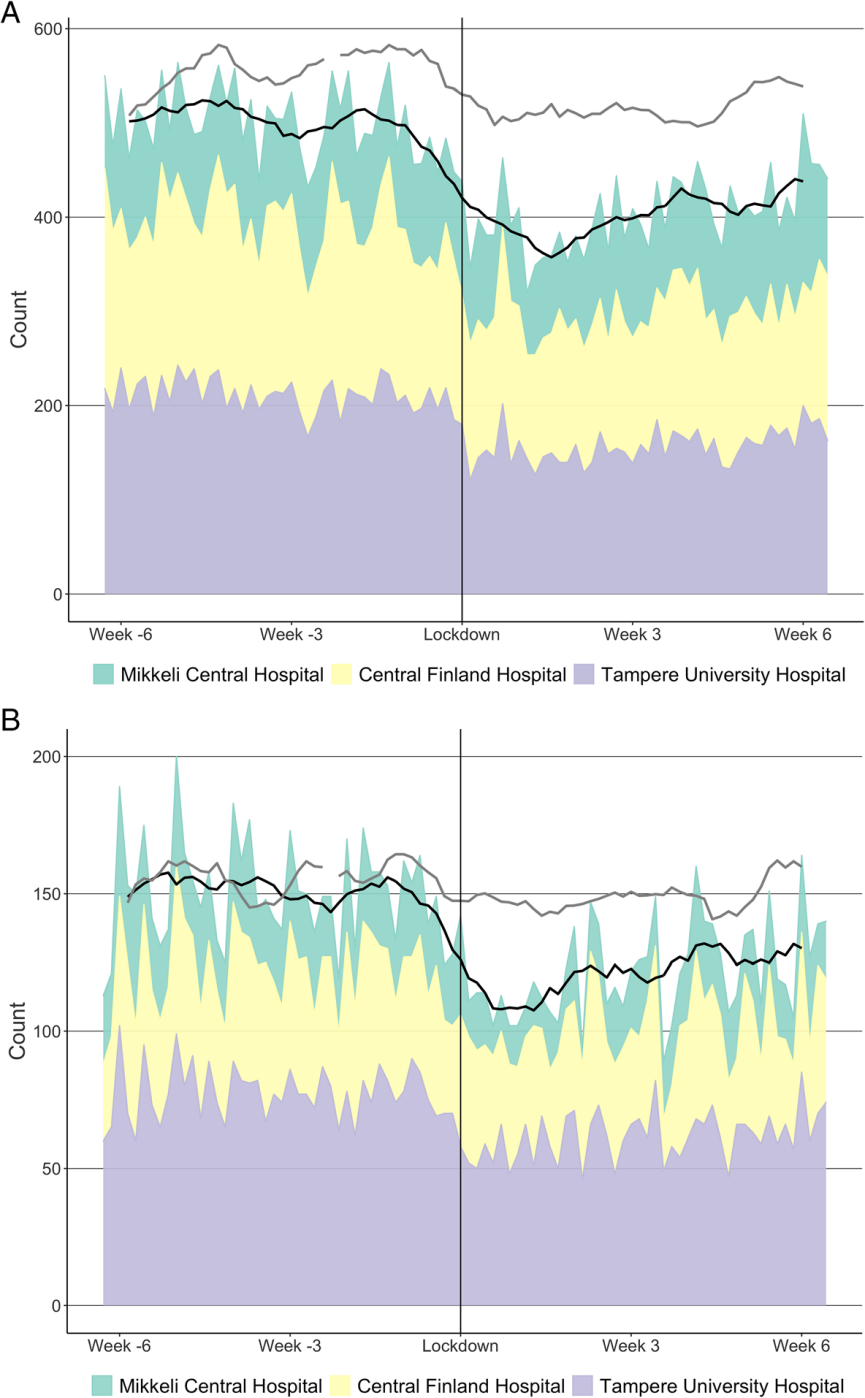
All visits to emergency departments (**a**), inpatient admissions from emergency department (**b**) and emergency six weeks before and after the declaration of the state of emergency. Black line presents weekly mean in 2020 and grey line weekly mean during the corresponding time period in 2019

The total number of ED visits during the 12-week study period was 40,653. Of these visits, 22,069 (54%) occurred before the lockdown and 18,584 (46%) during the lockdown. After the announcement of the lockdown, the daily median number of ED visits decreased notably in TAUH and CFH, but remained stable in MCH (Table 1). The rolling mean of weekly visits in all participating hospitals showed a notable drop immediately after the declaration of the lockdown. A similar drop was not, however, seen in the reference year 2019 (Fig. 1a).

**Table 1:** Number of emergency department (ED) visits and inpatient admissions in the participating hospitals six weeks before and six weeks after the start of emergency on March 16

	six weeks before		six weeks after		
	median	IQR	median	IQR	p
Combined					
total n of ED visits	22,069		18,584		
total n of inpatient admissions^a^ (%)	6606	29.9	5620	30.2	0.50
Tampere University Hospital					
daily median of ED visits	213	29	155	24	< 0.001
daily median of inpatient admissions	77	15	61	13	< 0.001
Central Finland Hospital					
daily median of ED visits	180	43	141	31	< 0.001
daily median of inpatient admissions	44	16	40	15	0.02
Mikkeli Central Hospital					
daily median of ED visits	102	20	101	24	0.18
daily median of inpatient admissions	26	8	21	9	< 0.001

^a^Chi square test was used

**Fig. 2:**
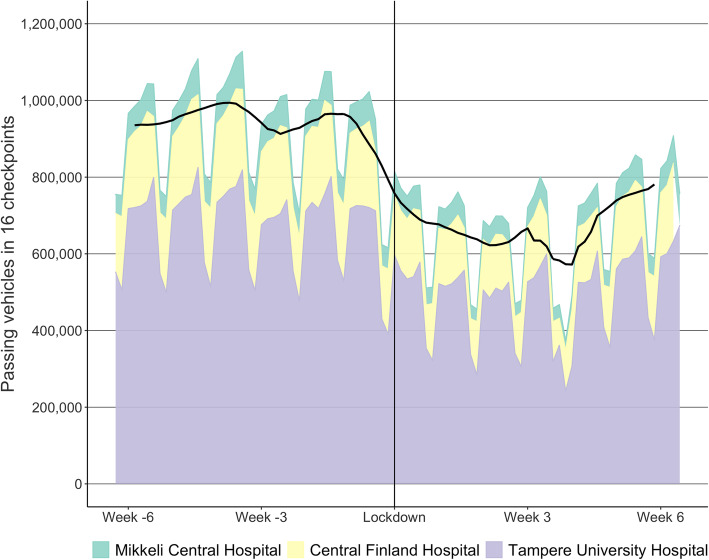
The decrease in general activity illustrated by the volume of traffic on the main roads of the catchment areas of the three study hospitals six weeks before and after the declaration of the state of emergency. Black line presents weekly mean

The total number of inpatient admissions during the 12-week study period was 12,226, and of these, 6606 (54%) occurred before and 5620 (46%) during the lockdown. The most prominent decrease in inpatient admissions was seen to start rapidly a week before the lockdown and this decreasing trend continued until the first week of the lockdown. Thereafter, the rate of inpatient admissions remained low. This downward trend in hospital admissions was not seen in the corresponding time period in 2019 (Fig. 1b). The rate of inpatient admissions decreased in all participating hospitals (Table 1). The decreasing trends in daily ED visits and inpatient admissions from the ED reflected similar trends seen in general mobility in the catchment areas of the participating hospitals (Fig. 2).

**Fig. 3:**
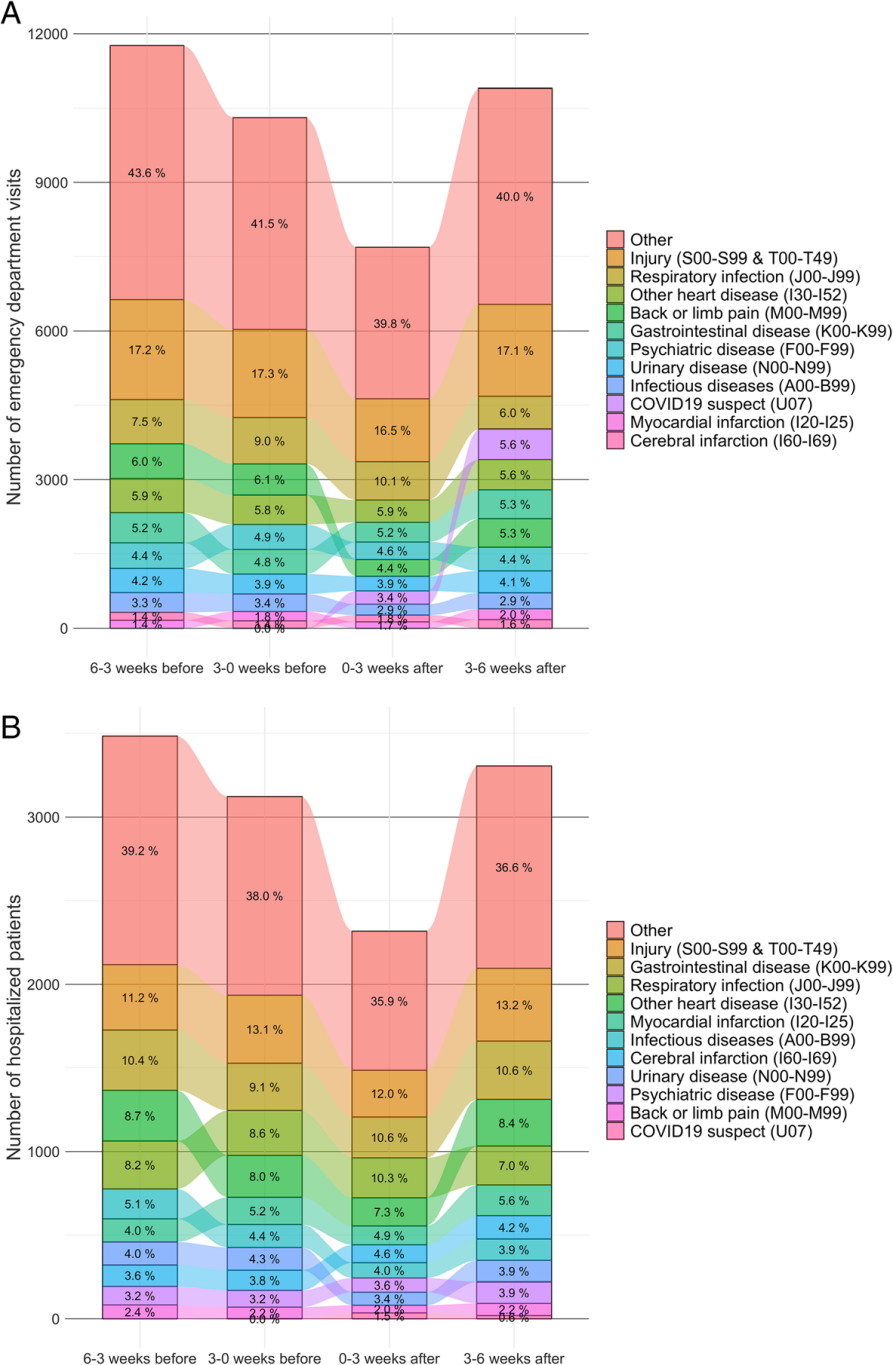
The most common diagnoses and reasons of **a**) visits to emergency departments, **b**) inpatient admissions from ED in three-week periods starting six weeks before the state of emergency and ending six weeks after the state of emergency on March 16, 2020. Visits with missing diagnoses excluded

The diagnoses of ED patients varied during the study period. The most common diagnostic group before and during the lockdown was injuries and traumas (ICD-10 S00-S99 & T00-T49), followed by respiratory diseases (J00-J99) (Fig. 3a). During the lockdown, the number of visits due to back or limb pain (M00-M99) decreased the most (− 31%). Interestingly, the number of visits due to other heart diseases (I30-I52) also decreased (− 17%). The rates of acute myocardial infarctions (I20-I25) and strokes (I60-I69) remained stable throughout the study period (Table 2).

**Table 2:** Numbers and change in given diagnoses in emergency department visits in the participating hospitals six weeks before and six weeks after the start of emergency on March 16

	six weeks before	six weeks after	
	n	n	Change (%)
Total number of visits	22,069	18,584	−15.8
Visits due:			
-Back or limb pain (M00-M99)	1333	916	−31.3
-Cerebral infarction (I60-I69)	308	311	1.0
-COVID19 suspect (U07)^a^	2	262	13,000
-Gastrointestinal disease (K00-K99)	1111	981	−11.7
-Infectious diseases (A00-B99)	741	537	−27.5
-Injury (S00-S99 & T00-T49)	3804	3128	−17.8
-Myocardial infarction (I20-I25)	348	348	0.0
-Other	9404	8030	−14.6
-Other heart disease (I30-I52)	1284	1066	−17.0
-Psychiatric disease (F00-F99)	1024	834	−18.6
-Respiratory infection (J00-J99)	1818	1431	−21.3
-Urinary disease (N00-N99)	892	740	−17.0

^a^ICD-10 code U07.1 was introduced in Finland on March 11, meaning that the visits due to suspected or diagnosed COVID-19 infection were coded to different categories prior to its introduction

The reasons for inpatient admissions varied before and during the lockdown. The most common reasons for inpatient admissions during the lockdown were traumas (S00-S99 & T00-T49), respiratory diseases (J00-J99), and other heart diseases (I30-I52). (Fig. 3b) The decrease was most prominent in admissions due to infectious diseases (A00-B99) with the rate of − 31%, urinary diseases (N00-N99) with a rate of − 25%, and back or limb pain (M00-M99) with a rate of − 22%. The rates of inpatient admissions due to strokes (I60-I69), acute myocardial infarctions, and psychiatric diseases (F00-F99) remained stable during the lockdown (Table 3).

**Table 3:** Numbers and change in given diagnoses of inpatient admissions in emergency departments in the participating hospitals six weeks before and six weeks after the start of lockdown on March 16

	six weeks before	six weeks after	
	n	n	Change (%)
Total number of inpatient admissions	6606	5620	−14.9
Inpatient admissions due:			
-Back or limb pain (M00-M99)	152	119	−21.7
-Cerebral infarction (I60-I69)	245	247	0.8
-COVID19 suspected or detected (U07) ^a^	1	54	5300.0
-Gastrointestinal disease (K00-K99)	644	594	−7.8
-Infectious diseases (A00-B99)	317	220	−30.6
-Injury (S00-S99 & T00-T49)	800	714	−10.8
-Myocardial infarction (I20-I25)	302	297	−1.7
-Other	2553	2039	−20.1
-Other heart disease (I30-I52)	553	447	−19.2
-Psychiatric disease (F00-F99)	211	211	0.0
-Respiratory infection (J00-J99)	554	472	−14.8
-Urinary disease (N00-N99)	274	206	−24.8

^a^ICD-10 code U07.1 was introduced in Finland on March 11, meaning that the visits due suspected or diagnosed COVID-19 infection were coded to different categories prior to its introduction

## Discussion

Very soon after the lockdown was declared, the total number of daily ED visits and inpatient admissions from ED decreased markedly in all of the participating hospitals.

The advice issued to citizens by the Finnish Institute of Health and Welfare concerning the lockdown was to treat minor diseases and symptoms at home and to visit the ED only after first contacting the ED by phone [16]. This advice might be one of the reasons for the decrease in visits. Another reason might be a reluctance to go to hospital during the state of emergency because of a fear of contracting COVID-19 during in-hospital stay. However, it should be noted that the number of patients with COVID-19 in the study hospitals had remained low [17].

Although a notable decrease in patient numbers occurred among other hearth diseases, such as atrial fibrillation and heart failures, our results suggest that patients with severe diseases, such as acute myocardial infarction, still sought out ED’s during the lockdown. The Finnish Heart Association published a statement for patients with heart conditions to seek treatment during these exceptional times and not to fear visiting hospital when needed [18], and this may have had an effect on patient numbers.

The authors were unable to find previously published literature on lockdowns and the use of emergency departments and hospital resources. The rationale behind the lockdown is that social distancing measures should decrease the spread of infections [9, 19, 20]. Correspondingly, we found that the number of visits and inpatient admissions due to respiratory infections and infectious diseases decreased during the lockdown. Also, the fear of COVID-19 and recommendations to stay at home with mild symptoms has also likely affected these trends. In MCH the rate of ED visits remained nearly unchanged during the lockdown. As the lockdown started all patients with infectious diseases in MCH catchment area were guided to MCH ED. Usually these patients may visit occupational health, private sector and smaller primary healthcare units outside the hospital ED. This finding might explain the difference in comparison to CFH and TAUH.

A more surprising finding was the overall decrease in the rate of hospital inpatient admissions from EDs. The decrease in inpatient admissions due to infectious diseases and respiratory infections might be explained by social distancing reducing the spread of common viral infections. However, the rate of admissions due to strokes and acute myocardial infarctions remained stable, which may indicate that patients in real need still visit ED’s. The decrease in admissions was also most notable among patients with back or limb pain, urinary diseases, and other heart diseases. Based on these findings, it might be speculated that some of these diseases might not actually need to be treated in ED’s at all, and whether all the previous inpatient admissions were actually needed. Another possibility is that the COVID-19 pandemic has created a “treatment loan” that we will have to take care of in the future. In this present study, the rate of psychiatric inpatient admissions remained stable during the lockdown, although the actual ED visit rate of these patients decreased during the study period. This finding indicates that severe cases in need of hospital care should be properly identified in psychiatric hospitals.

During April, the growth of COVID-19 in Finland cases declined and the number of hospitalized patients and deaths stabilized (a total of 200 hospitalized patients and 5 to 10 deaths daily) [17]. In the participating hospitals, the number of patients hospitalized with COVID-19 has been low (a total of 54 patients during the 6-week study period). However, it is not certain how precisely people obey the restrictions. As trends in traffic activity has started to return to normal, a similar increasing trend in visits to EDs and hospital admissions has also been seen.

One of the main strengths of this study is that the data were collected from Finland, a country with a public and practically free healthcare system. Our three study hospitals with centralized EDs cover 1/6 of the Finnish population, and our data included all patients that required hospitalization in these three hospital catchment areas. A weakness of the study was that we were only able to include rates from one previous year as a reference. However, we used data from the Finnish Transport Infrastructure Agency to represent general mobility to have a reference of the overall mobility of the participating areas. Also, it must be noted that the COVID-19 diagnosis U07.1 was first introduced during our study period, and it was first used for both suspected and laboratory diagnosed cases. Therefore, it may not completely correlate with the real number of reported patients in our ED’s. The diagnosis code U07.2 was introduced and implemented in April, 2020. Our study counts both U07.1 and U07.2 under the diagnosis coding U07.

## Conclusions

Changes in the numbers of ED visits and inpatient admissions prior to and during the early phase of the COVID-19 outbreak were unpredictable and our results may help hospitals and especially ED’s focus their resources better. Surprisingly, there was a major decrease in the rate of ED visits due to back or limb pain and not so surprisingly in infectious diseases. The rates of acute myocardial infarctions and cerebral strokes remained stable. In summary, stabile resources for the treatment of patients with severe diseases will always be needed in hospitals and ED’s.

## Data Availability

Please contact author for data requests.

## Abbreviations

CFH: Central Finland Hospital
ED: Emergency department
MCH: Mikkeli Central Hospital
TAUH: Tampere University Hospital

## References

[1] Huang C, Wang Y, Li X, Ren L, Zhao J, Hu Y (2020). Clinical features of patients infected with 2019 novel coronavirus in Wuhan, China. Lancet.

[2] Khan S, Siddique R, Shereen MA, Ali A, Liu J, Bai Q, et al. The emergence of a novel coronavirus (SARS-CoV-2), their biology and therapeutic options. J Clin Microbiol. 2020;11. 10.1128/JCM.00187-20 PMC7180238 32161092

[3] Lai C, Shih T, Ko W, Tang H, Hsueh P (2020). Severe acute respiratory syndrome coronavirus 2 (SARS-CoV-2) and coronavirus disease-2019 (COVID-19): the epidemic and the challenges. Int J Antimicrob Agents.

[4] Zhao S, Lin Q, Ran J, Musa SS, Yang G, Wang W (2020). Preliminary estimation of the basic reproduction number of novel coronavirus (2019-nCoV) in China, from 2019 to 2020: a data-driven analysis in the early phase of the outbreak. Int J Infect Dis.

[5] WHO announces COVID-19 outbreak a pandemic [Internet].: World Health Organization; 2020 [updated −03-12; cited Mar 22, 2020]. Available from: http://www.euro.who.int/en/health-topics/health-emergencies/coronavirus-covid-19/news/news/2020/3/who-announces-covid-19-outbreak-a-pandemic.

[6] Finland's first coronavirus case confirmed in Lapland [Internet].; 2020 [updated Jan 29; cited Mar 22, 2020]. Available from: https://yle.fi/uutiset/osasto/news/finlands_first_coronavirus_case_confirmed_in_lapland/11182855.

[7] Infectious Disease Register [Internet].; 2020 [updated Mar 22; cited Mar 22 2020]. Available from: https://thl.fi/en/web/infectious-diseases/surveillance/infectious-disease-register.

[8] Preparedness for the novel coronavirus disease - A state of emergency in Finland [Internet].; 2020 [updated Mar 16,; cited Mar 19, 2020]. Available from: https://stm.fi/en/fighting-infectious-disease.

[9] Wilder-Smith A, Freedman DO. Isolation, quarantine, social distancing and community containment: pivotal role for old-style public health measures in the novel coronavirus (2019-nCoV) outbreak. J Travel Med. 2020;27(2). 10.1093/jtm/taaa020 PMC7107565 32052841

[10] Ahmed F, Zviedrite N, Uzicanin A (2018). Effectiveness of workplace social distancing measures in reducing influenza transmission: a systematic review. BMC Public Health.

[11] Roosa K, Lee Y, Luo R, Kirpich A, Rothenberg R, Hyman JM, Yan P, Chowell G. Short-term Forecasts of the COVID-19 Epidemic in Guangdong and Zhejiang, China: February 13–23, 2020. J Clin Med. 2020;9(2):596. 10.3390/jcm9020596 PMC7073898 32098289

[12] Remuzzi A, Remuzzi G. COVID-19 and Italy: what next? Lancet. 2020. 10.1016/S0140-6736(20)30627-9 PMC7102589 32178769

[13] Choi S, Ki M. Estimating the reproductive number and the outbreak size of COVID-19 in Korea. Epidemiol Health. 2020;42:e2020011. 10.4178/epih.e2020011 Online first. 10.4178/epih.e2020011 PMC7285447 32164053

[14] Population Information System, Finnish inhabitants 2019. Statistics Finland. [Internet]. 2020 [updated 31.12.2019,; cited Mar 19, 2020]. Available from: https://dvv.fi/en/population-information-system.

[15] Road statistics - Open Access Statistical Report [Internet]. [cited Apr 14, 2020]. Available from: http://vayla.fi/web/en/statistics/road-statistics.

[16] Guidelines for citizens regarding to COVID-19 [Internet].; 2020 [updated Mar 16,; cited Mar 19, 2020]. Available from: https://thl.fi/fi/web/infektiotaudit-ja-rokotukset/ajankohtaista/ajankohtaista-koronaviruksesta-covid-19/ohjeita-kansalaisille-koronaviruksesta.

[17] Situation update on coronavirus - Infectious diseases - THL [Internet]. [cited Apr 17, 2020]. Available from: https://thl.fi/en/web/infectious-diseases/what-s-new/coronavirus-covid-19-latest-updates/situation-update-on-coronavirus.

[18] Tärkeitä ohjeita sydänoireisiin ja tutkimuksiin liittyen koronavirusepidemian aikana - Acute Myocardial Ischemias during COVID-19 epidemic [Internet].; 2020 [updated Apr 22; cited Apr 28, 2020]. Available from: https://sydan.fi/fakta/tarkeita-ohjeita-sydanoireisiin-ja-tutkimuksiin-liittyen-koronavirusepidemian-aikana/.

[19] Pan A, Liu L, Wang C, Guo H, Hao X, Wang Q, et al. Association of Public Health Interventions With the Epidemiology of the COVID-19 Outbreak in Wuhan, China. JAMA. 2020. 10.1001/jama.2020.6130 PMC7149375 32275295

[20] Sakamoto H, Ishikane M, Ueda P. Seasonal Influenza Activity During the SARS-CoV-2 Outbreak in Japan. JAMA. 2020;323(19):1969-71. 10.1001/jama.2020.6173 PMC7149351 32275293

